# Timing Deficits in ADHD: Insights From the Neuroscience of Musical Rhythm

**DOI:** 10.3389/fncom.2018.00051

**Published:** 2018-07-06

**Authors:** Jessica L. Slater, Matthew C. Tate

**Affiliations:** ^1^Department of Neurological Surgery, Northwestern University, Chicago, IL, United States; ^2^Department of Neurology, Northwestern University, Chicago, IL, United States

**Keywords:** music, rhythm, attention deficit hyperactivity disorder, ADHD, cognitive control, motor timing, neuroplasticity, musical expertise

## Abstract

Everyday human behavior relies upon extraordinary feats of coordination within the brain. In this perspective paper, we argue that the rich temporal structure of music provides an informative context in which to investigate how the brain coordinates its complex activities in time, and how that coordination can be disrupted. We bring insights from the neuroscience of musical rhythm to considerations of timing deficits in Attention Deficit/Hyperactivity Disorder (ADHD), highlighting the significant overlap between neural systems involved in processing musical rhythm and those implicated in ADHD. We suggest that timing deficits warrant closer investigation since they could lead to the identification of potentially informative phenotypes, tied to neurobiological and genetic factors. Our novel interdisciplinary approach builds upon recent trends in both fields of research: in the neuroscience of rhythm, an increasingly nuanced understanding of the specific contributions of neural systems to rhythm processing, and in ADHD, an increasing focus on differentiating phenotypes and identifying distinct etiological pathways associated with the disorder. Finally, we consider the impact of musical experience on rhythm processing and the potential value of musical rhythm in therapeutic interventions.

## Introduction

Music is pervasive across cultures and plays an important role in human interaction, development and social bonding (Cross, 2001). The temporal structure of music is integral to its functions, and the experience of music relies upon a precisely-timed orchestration of activity across the brain's sensory, cognitive, motor, and reward systems. Musical rhythms inspire us to move (Keller and Rieger, 2009; Dalla Bella et al., 2013), and movement can, in turn, shape our perception of rhythmic patterns (Phillips-Silver and Trainor, 2005, 2007). Music also facilitates interpersonal synchrony, increasing pro-social behavior (Cirelli et al., 2012, 2014) and breaking down perceived barriers between self and other by coordinating shared emotional experiences (Tarr et al., 2014). Several studies suggest that interaction with music promotes synchronous neural activity not only across brain regions, but between the brains of individuals, for example during music listening (Abrams et al., 2013) and improvisation (Müller et al., 2013).

The rewarding qualities of music are also intrinsically linked to its temporal structure, through the creation and manipulation of expectations over time (Cooper and Meyer, 1960; Huron, 2006). Within this temporal framework, the fulfillment and violation of expectations provides a rich palette of emotional expression, mediated by the reward transmitter dopamine (Schultz, 1998; Salimpoor and Zatorre, 2013). The rhythmic patterns found across a range of musical styles have been shown to exhibit an optimal balance of predictability and surprise, even in their written form (Levitin et al., 2012), and the subtle timing variations found in live musical performance further contribute to the emotional expression perceived by a listener (Repp, 1995; Palmer, 1997; Ashley, 2002; Bhatara et al., 2011). As these examples highlight, the influence of music on human experience is closely tied to its temporal structure and the coordinated neural activity it induces, both within and between individuals.

The dynamic interplay between predictive (top-down) and reactive (bottom-up) processing, exemplified in how the brain responds to musical rhythm, is also a necessary foundation for cognitive functions, such as attention (Engel et al., 2001; Raichle, 2010). For example, the ability to anticipate what is likely to happen next and streamline the allocation of neural resources accordingly must be balanced with the ability to respond to unexpected salient events in the environment. In disorders such as ADHD, this balance is disrupted, resulting in impaired attentional control and difficulties inhibiting irrelevant inputs. We have chosen to consider ADHD in particular because in addition to the core symptoms of inattention and/or hyperactivity/impulsivity, ADHD is also characterized by deficits in motor and perceptual timing (Smith et al., 2002; Fair et al., 2012; Zelaznik et al., 2012; Demers et al., 2013; Noreika et al., 2013). Recent studies have revealed rhythm-related deficits in ADHD (Hove et al., 2017; Puyjarinet et al., 2017), and much of the same neural infrastructure that supports the processing of musical rhythm is implicated in ADHD, from brain circuitry (Silk et al., 2009; Silberstein et al., 2016; Mueller et al., 2017) and neural dynamics (Başar and Güntekin, 2008; Mazaheri et al., 2014; Loo et al., 2017) to dopamine signaling, with leading genetic risk factors for ADHD including dopamine gene variants (Swanson et al., 2000; DiMaio et al., 2003). Here, we propose that insights from research on musical rhythm could offer a more nuanced understanding of timing deficits in ADHD, and potentially lead to the identification of informative phenotypes, linked to neurobiological and genetic factors.

## The neural infrastructure of musical rhythm

In this section we highlight key components of the neural infrastructure involved in processing musical rhythm. Although this is by no means an exhaustive review, some basic definitions of terms may prove useful. We will use the term “rhythm” to refer to temporal patterns formed from sequences of durations or onsets, whereas “beat” refers to a periodic pulse. In a piece of music, the beat typically defines the basic unit of timing, and “meter” refers to the grouping of beats into a recurring pattern of stresses or accents, such as would differentiate the feel of a waltz vs. a march.

### Sensory-motor integration

Studies with non-human primates and even zebrafish have shown that neural ensembles can entrain to a rhythmic stimulus (Quintana and Fuster, 1999; Sumbre et al., 2008), and it is likely that human interaction with musical rhythm is founded upon these basic entrainment mechanisms. However, it is notable that the natural human tendency to move to music, for example by tapping a foot to the beat, has proven surprisingly elusive in the animal kingdom (Patel et al., 2009).

Imaging studies have revealed that in humans, rhythm perception is associated with activation not only in auditory cortices but in frontal, parietal and motor regions, including the supplementary motor area (SMA), basal ganglia and cerebellum (Grahn and Brett, 2007; Grahn, 2012; Large et al., 2015; Merchant et al., 2015). It has been suggested that the close sensory-motor coupling necessary for synchronization of movement to music may be unique to vocal learning species (including parrots and songbirds, as well as humans), in which it is a necessary basis for learning and producing complex communication signals (Patel and Iversen, 2014). Recent evidence of successful entrainment to the musical beat in non-vocal-learning species, for example a California sea lion (Cook et al., 2013), have cast doubt on this theory. Nonetheless, it is well established that close interaction between sensory and motor systems provides a sophisticated mechanism of temporal prediction and feedback (Schroeder et al., 2010), and that this plays an important role in how humans process musical rhythm.

The extensive activation of motor areas during rhythm perception, even in the absence of overt movement (Zatorre et al., 2007; Chen et al., 2008; Grahn and Rowe, 2009), is consistent with accumulating evidence that these systems serve a broader role in temporal processing and cognition. For example, fronto-striatal and fronto-cerebellar pathways are increasingly viewed as contributing to more general pattern-detection, predictive and cognitive functions (Akshoomoff and Courchesne, 1992; Graybiel, 1997; Schubotz, 2007). It has been proposed that striatal pathways are particularly involved in generating internal representations of beat and metrical structure (Grahn and Brett, 2007; Schwartze and Kotz, 2013). On the other hand, cerebellar circuits are more involved in the precise encoding of complex sequences, fast timing features and durations (Grube et al., 2010; Schwartze and Kotz, 2013). Together, these pathways create a system that can generate complex temporal predictions while also adapting to incoming information.

### Models of rhythm perception

In constructing computational models of rhythm perception, a major challenge is to capture not only the individual components of temporal processing that are involved, but how those mechanisms interact in real time to maintain the ongoing balance between predictive (top-down) and reactive (bottom-up) processing, discussed above (see McAuley, 2010; Grahn, 2012, for review). For example, several rule-based models have been proposed in which the regular beat and metrical structure inferred by a rhythmic pattern are maintained by an internal clock (Longuet-Higgins and Lee, 1982; Povel and Essens, 1985; Desain and Honing, 1999). However, these models do not generally account for adaptive, online predictions and instead determine a “best fit” pattern of regular intervals based on the rhythm sequence as a whole (summarized in Grahn, 2012).

Models based on the entrainment of multiple oscillators have had greater success in accounting for online prediction that is tolerant to more complex rhythmic structure while remaining sensitive to natural variations in performance (Large and Kolen, 1994; Large and Palmer, 2002; Angelis et al., 2013). Indeed, there is evidence to suggest that natural, non-random patterns of timing variability (i.e., those exhibiting fractal scaling and long-range correlations) may actually improve the accuracy of listeners' temporal predictions (Rankin et al., 2009, 2014), and this was also demonstrated by the model (Large and Palmer, 2002).

In their theory of neural resonance, Large and Snyder extend these computational models to propose that entrainment is performed in the brain by neural oscillators (Large and Snyder, 2009), and this theory is supported by evidence from imaging and EEG studies (Large and Snyder, 2009; Nozaradan et al., 2012; Tierney and Kraus, 2015). Interestingly, individual variation in the temporal characteristics of neural activity (including long-range correlations) has been shown to predict variability in motor timing behavior (Linkenkaer-Hansen et al., 2001; Smit et al., 2013). A recent paper also linked these temporal characteristics of neural activity to fluctuations in attention, and it was proposed that the typical increase in long-range correlations over the course of development may be delayed or disrupted in ADHD (Smit and Anokhin, 2017). This represents a fascinating area for future study, and a further potential link between ADHD and the temporal dynamics of brain and behavior.

Within entrainment models, different frequencies of neural oscillations serve distinct functions. For example, Large and Snyder suggest that bursts of high frequency oscillatory activity facilitate coordination across motor and sensory systems. Peaks in beta (13–30 Hz) and gamma (30–100 Hz) power were observed as an anticipatory response to rhythmic patterns (Snyder and Large, 2005; Fujioka et al., 2009), and persisted even when the sound stimulus stopped, supporting their role as self-sustaining timekeepers. Further, temporal modulations in beta activity were altered by the specific metrical structure imposed by the listener onto an ambiguous rhythm pattern, suggesting top-down modulation of oscillatory dynamics (Iversen et al., 2009). Given the association between beta oscillations and motor coordination, the modulation of beta power may provide another indication of the influence of motor systems on rhythm processing (Large et al., 2015).

Neural responses to musical rhythm may also take the form of entrainment to specific frequencies actually present in the stimulus, for example the frequency of the musical beat. Neural entrainment to the beat has been observed in a number of EEG studies in the form of increased spectral power at the frequency corresponding to the tempo of the musical beat, typically within the delta range (1–4 Hz), and even to harmonics and subharmonics of that frequency (Nozaradan et al., 2012; Tierney and Kraus, 2013, 2015; Nozaradan, 2014). The influence of motor systems on this form of neural beat entrainment was investigated in a recent lesion study (Nozaradan et al., 2017). Both cerebellar and basal ganglia patients showed reduced neural activity aligned with the beat compared with controls, with cerebellar patients showing reductions specifically with faster tempo rhythms, and basal ganglia patients showing a greater deficit with complex rhythm patterns, which the authors interpreted as relying more heavily on the internal generation of a beat. These findings suggest that variation in cerebellar and striatal function (such as observed in ADHD) may be associated with distinct rhythm processing deficits. This study therefore provides compelling evidence for distinct specializations of these two motor areas in the coordination of neural entrainment to musical rhythm, linked with dissociable deficits.

## Parsing heterogeneity in ADHD: the search for phenotypes

ADHD is a highly prevalent and heterogenous disorder. Despite significant research efforts, characterization of the neurobiological basis of ADHD has proven elusive: diagnosis still relies heavily on self-report questionnaires, and treatment typically takes the form of a trial-and-error pharmacological approach. It has been difficult to identify biomarkers of the disorder because there has been no clear mapping between neural measures and clinical subtypes (i.e., predominantly inattentive, predominantly hyperactive/impulsive and combined type).

Although ADHD is associated with structural and functional abnormalities, including within frontal, striatal and cerebellar pathways, these findings have generally been small, and have not always been replicated (see Rubia, 2016, for review). Similarly, profiles of oscillatory dynamics have not been consistent enough to provide a clear neural “signature” of ADHD. EEG studies reveal abnormal patterns of oscillatory activity (Başar and Güntekin, 2008; Mazaheri et al., 2014; Loo et al., 2017), including reduced power in the beta frequency range. Indeed, a clinical diagnostic device assessing the ratio between theta and beta activity was developed and approved by the FDA (USDHHS, 2013). However, a subsequent meta-analysis suggested the theta-beta ratio is only elevated within a subgroup of individuals with ADHD, and is therefore not a reliable basis for diagnosis (Arns et al., 2013). A more nuanced understanding of distinct phenotypes of ADHD could help to increase diagnostic accuracy, and improve the development of clinical tools to aid in the evaluation and monitoring of treatment.

Research in the field is shifting toward the identification of distinct phenotypes and multiple etiologies (Castellanos and Tannock, 2002; Nigg et al., 2005; Durston et al., 2011). There is evidence from neuropsychological (Rommelse et al., 2008; Fair et al., 2012; Nikolas and Nigg, 2015), electrophysiological (Başar and Güntekin, 2008; Mazaheri et al., 2014; Loo et al., 2017) and genetic studies (Shaw et al., 2007; Giedd et al., 2008; Kebir and Joober, 2011) to suggest the presence of distinct subgroups within ADHD, beyond the clinical subtypes. However, these subgroups have yet to be reconciled across methodologies to provide full characterization of etiological pathways.

Although motor and timing deficits are not included within the diagnostic criteria for ADHD, they are increasingly recognized as common symptoms (Toplak et al., 2006; Demers et al., 2013; Kaiser et al., 2015; Dahan et al., 2016), and have been identified as a promising area for future study (Rubia, 2016). Consistent with the presence of multiple phenotypes, a recent study identifying rhythm deficits in children and adults with ADHD noted significant variation in performance within the ADHD group (Puyjarinet et al., 2017). Based on neuropsychological studies, it has been suggested that deficits in temporal information processing (e.g., duration discrimination) and increased response variability may represent distinct phenotypes, linked to dysfunction in cerebellar and basal ganglia pathways, respectively (Durston et al., 2011; Fair et al., 2012). Given the distinct roles of fronto-cerebellar and fronto-striatal pathways in rhythm processing (Grahn and Brett, 2009; Grahn, 2012; Merchant et al., 2015; Nozaradan et al., 2017), including their separate influence on neural entrainment discussed in the previous section, we argue that further examination of rhythm-related deficits in ADHD could help to characterize phenotypes of ADHD, and to shed light on the different ways in which the dynamics within associated neural systems may be disrupted.

Further, genetic risk factors for ADHD include genes affecting dopaminergic transmission, which may influence timing behavior (Valera et al., 2010). This is supported by pharmacological studies in which timing deficits in ADHD are reduced by methylphenidate (which increases levels of dopamine) (Noreika et al., 2013) as well as a study in which dopamine manipulation in healthy controls was associated with impaired timing skills (Coull et al., 2012). As mentioned in the introduction, dopamine indexes temporal expectation within the context of musical rhythm. More broadly, dopamine supports neural communication within reward, motor and cognitive pathways and is involved in a wide range of functions including reward-based learning, motor coordination and cognitive control. It has been proposed that a common theme across its various functions is that dopamine coordinates neural systems to optimize responsiveness at different timescales, matching the timescales of activity in the environment (Schultz, 2007). In other words, dopamine helps to keep the brain “in sync” with the world around it. This is accomplished via multiple dopamine release mechanisms with distinct kinetic properties (Schultz, 2007). Therefore, we speculate that genetic variation in specific components of the dopaminergic system could lead to distinct deficits in neural and behavioral timing. This is consistent with evidence from animal studies, in which different genetic modifications affecting dopamine transmission in mice were associated with distinct behavioral timing deficits (Cevik, 2003; Drew et al., 2007; Balci et al., 2009, 2010), as well as evidence of dissociable timing deficits in humans linked to dopamine gene variants (Wiener et al., 2011).

Dopamine also helps to mediate the balance between inhibitory and excitatory neural activity that sustains neural oscillations, therefore genetic variations in dopaminergic signaling at different timescales may also influence temporal characteristics of oscillatory dynamics, such as the long-range correlations discussed above. Disrupted neural dynamics may in turn influence the development of cortical networks (Uhlhaas et al., 2010). Indeed, longitudinal studies have demonstrated distinct trajectories of structural brain development associated with different dopamine gene polymorphisms in ADHD (Shaw et al., 2007; Giedd et al., 2008), however the potential role of neural dynamics in mediating these developmental differences remains to be explored. Recent research indicates that ADHD, neural dynamics and timing-related behaviors are all heritable (Tye et al., 2011; Agostino and Cheng, 2016), suggesting that a “genes to behavior” approach may prove fruitful.

## Effects of expertise

Several aspects of rhythm processing that are implicated in ADHD are also strengthened in expert musicians (summarized in Table 1), suggesting the potential for these systems to be shaped by experience. Behaviorally, musicians are better than controls at rhythm perception and temporal discrimination tasks (Rammsayer and Altenmüller, 2006; Wallentin et al., 2010) and have more consistent sensorimotor timing (Repp and Su, 2013). They also demonstrate enhanced cognitive function, including attention, inhibitory control and working memory (see Benz et al., 2015, for recent review), with enhanced inhibitory control linked to more consistent sensorimotor timing (Slater et al., 2017, 2018). Researchers found that musicians had larger volumes in motor areas including the cerebellum and basal ganglia, as well as frontal and parietal regions associated with cognitive control (see Schlaug, 2015, for review), and music training has been associated with functional changes to oscillatory dynamics (Bhattacharya and Petsche, 2005; Trainor et al., 2009).

**Table 1:** How the infrastructure of musical rhythm processing is influenced by ADHD and musical expertise.

	**Cognitive function**	**Sensorimotor timing**	**Rhythm perception**	**Neural dynamics**	**Neural pathways**	**Neuromodulatory systems**
ADHD	Deficits in attention, inhibitory control, working memory.	Increased motor timing variability, linked to poor inhibitory control.	Difficulties with beat perception and duration estimation.	Abnormal patterns of oscillatory activity across multiple frequency bands.	Decreased volumes in frontal, parietal and motor regions, including cerebellum and basal ganglia. Decreased connectivity within motor and cognitive control networks.	Disrupted dopaminergic signaling, linked to genetic variation in dopamine receptors and transporters.
Musicians	Enhanced attention, inhibitory control, working memory.	More consistent sensorimotor timing, correlated with enhanced inhibitory control.	Improved accuracy in beat perception and duration discrimination tasks.	Functional changes in oscillatory activity linked to music training, including increased coherence between frequencies.	Increased cerebellar and basal ganglia volumes. Increased connectivity within motor and cognitive control networks.	Preliminary evidence for increased dopamine receptor expression in musicians (potentially indicating genetic predisposition to music).

It is possible that group comparisons reflect innate differences in those drawn to pursue music rather than causal effects of training, in fact there is some preliminary evidence showing increased expression of dopamine receptors in musicians compared with controls, suggesting a potential genetic tendency toward musicianship (Emanuele et al., 2010). However, evidence from longitudinal studies (Moreno et al., 2011; Roden et al., 2014) as well as links between behavioral enhancements, extent of expertise (Slater et al., 2018) and specific instrument played (Krause et al., 2010) suggest that experience plays at least some role in observed differences. Further, therapies focusing on motor timing or rhythm have shown some success in ameliorating the broader symptoms of ADHD (Shaffer et al., 2001; Leisman and Melillo, 2010; Dahan et al., 2016), although more intervention studies are needed. Taken together, these findings suggest that common underlying mechanisms involved in both cognitive and motor control could potentially be strengthened by music-based interventions, building on the established use of music-based therapies in the treatment of a variety of other disorders. With a clearer understanding of distinct phenotypes, the efficacy of such interventions for ADHD could be greatly improved.

## Conclusions

By considering how the brain processes musical rhythm, we force ourselves to take an integrated approach to how the brain coordinates its activities in time. Here, we argue that it is exactly this kind of integrated approach that is needed to advance understanding of a complex, heterogeneous disorder such as ADHD.

Whereas a great deal of neuroscientific research has focused on the spatial dimension—within perception itself, as well as in the localization of functions to particular brain regions—the inherently temporal nature of musical sound helps to bring mechanisms of neural coordination to the forefront. In this review, we have explored common neural infrastructure that is involved in processing musical rhythm, and implicated in ADHD. We have discussed how the heterogeneity of ADHD has hampered progress toward the identification of biomarkers and objective diagnostic tools. We suggest that further investigation of the basis of rhythm and timing deficits could ultimately help to form a more integrated view of the etiologies of ADHD, bridging the gap between genetic factors (e.g., variation in dopaminergic signaling), neural dynamics and the development of cortical networks, and the behavioral control of cognition and movement. We have also highlighted that the same neural systems are strengthened in expert musicians, suggesting the potential for neuroplasticity to have remediating effects. This novel, interdisciplinary approach could inform therapeutic strategies, harnessing the rewarding properties of music to strengthen coordination within the brain.

## Author contributions

JS conceived and wrote the article, MT contributed to the writing of the article.

### Conflict of interest statement

The authors declare that the research was conducted in the absence of any commercial or financial relationships that could be construed as a potential conflict of interest.

## References

[B1] Abrams D. A. Ryali S. Chen T. Chordia P. Khouzam A. Levitin D. J.. (2013). Inter-subject synchronization of brain responses during natural music listening. Eur. J. Neurosci. 37, 1458–1469. 10.1111/ejn.12173 23578016 PMC4487043

[B2] Agostino P. V. Cheng R.-K. (2016). Contributions of dopaminergic signaling to timing accuracy and precision. Curr. Opin. Behav. Sci. 8, 153–160. 10.1016/j.cobeha.2016.02.013

[B3] Akshoomoff N. A. Courchesne E. (1992). A new role for the cerebellum in cognitive operations. Behav. Neurosci. 106, 731. 10.1037/0735-7044.106.5.731 1445653

[B4] Angelis V. Holland S. Upton P. J. Clayton M. (2013). Testing a computational model of rhythm perception using polyrhythmic stimuli. J. New Music Res. 42, 47–60. 10.1080/09298215.2012.718791

[B5] Arns M. Conners C. K. Kraemer H. C. (2013). A decade of EEG theta/beta ratio research in ADHD: a meta-analysis. J. Atten. Disord. 17, 374–383. 10.1177/1087054712460087 23086616

[B6] Ashley R. (2002). Do [n't] change a hair for me: the art of jazz rubato. Music Percept. 19, 311–332. 10.1525/mp.2002.19.3.311

[B7] Balci F. Day M. Rooney A. Brunner D. (2009). Disrupted temporal control in the R6/2 mouse model of Huntington's disease. Behav. Neurosci. 123, 1353. 10.1037/a0017650 20001119

[B8] Balci F. Ludvig E. A. Abner R. Zhuang X. Poon P. Brunner D. (2010). Motivational effects on interval timing in dopamine transporter (DAT) knockdown mice. Brain Res. 1325, 89–99. 10.1016/j.brainres.2010.02.034 20167208

[B9] Başar E. Güntekin B. (2008). A review of brain oscillations in cognitive disorders and the role of neurotransmitters. Brain Res. 1235, 172–193. 10.1016/j.brainres.2008.06.103 18640103

[B10] Benz S. Sellaro R. Hommel B. Colzato L. S. (2015). Music makes the world go round: the impact of musical training on non-musical cognitive functions—a review. Front. Psychol. 6:2023. 10.3389/fpsyg.2015.02023 26779111 PMC4703819

[B11] Bhatara A. Tirovolas A. K. Duan L. M. Levy B. Levitin D. J. (2011). Perception of emotional expression in musical performance. J. Exp. Psychol. Hum. Percept. Perform. 37, 921. 10.1037/a0021922 21261418

[B12] Bhattacharya J. Petsche H. (2005). Phase synchrony analysis of EEG during music perception reveals changes in functional connectivity due to musical expertise. Signal Process. 85, 2161–2177. 10.1016/j.sigpro.2005.07.007

[B13] Castellanos F. X. Tannock R. (2002). Neuroscience of attention-deficit/hyperactivity disorder: the search for endophenotypes. Nature Rev. Neurosci. 3, 617–628. 10.1038/nrn896 12154363

[B14] Cevik M. (2003). Neurogenetics of interval timing, in Functional and Neural Mechanisms of Interval Timing, ed Meck W. H. (Boca Raton, FL: CRC Press), 297–316.

[B15] Chen J. L. Penhune V. B. Zatorre R. J. (2008). Listening to musical rhythms recruits motor regions of the brain. Cereb. Cortex 18, 2844–2854. 10.1093/cercor/bhn042 18388350

[B16] Cirelli L. K. Einarson K. Trainor L. J. (2012). Bouncing babies to the beat: Music and helping behaviour in infancy, in 12th International Conference on Music Perception and Cognition (Thessaloniki).

[B17] Cirelli L. K. Wan S. J. Trainor L. J. (2014). Fourteen-month-old infants use interpersonal synchrony as a cue to direct helpfulness. Philos. Trans. R. Soc. B Biol. Sci. 369:20130400. 10.1098/rstb.2013.0400 25385778 PMC4240967

[B18] Cook P. Rouse A. Wilson M. Reichmuth C. (2013). A California sea lion (Zalophus californianus) can keep the beat: motor entrainment to rhythmic auditory stimuli in a non vocal mimic. J. Comp. Psychol. 127, 412–27. 10.1037/a0032345 23544769

[B19] Cooper G. Meyer L. B. (1960). The Rhythmic Structure of Music. Chicago, IL: University of Chicago Press.

[B20] Coull J. T. Hwang H. J. Leyton M. Dagher A. (2012). Dopamine precursor depletion impairs timing in healthy volunteers by attenuating activity in putamen and supplementary motor area. J. Neurosci. 32, 16704–16715. 10.1523/JNEUROSCI.1258-12.2012 23175824 PMC6621775

[B21] Cross I. (2001). Music, cognition, culture, and evolution. Ann. N. Y. Acad. Sci. 930, 28–42. 10.1111/j.1749-6632.2001.tb05723.x 11458835

[B22] Dahan A. Ryder C. H. Reiner M. (2016). Components of motor deficiencies in ADHD and possible interventions. Neuroscience 378, 34–53. 10.1016/j.neuroscience.2016.05.040 27235737

[B23] Dalla Bella S. Białunska A. Sowinski J. (2013). Why movement is captured by music, but less by speech: role of temporal regularity. PLoS ONE 8:e71945. 10.1371/journal.pone.0071945 23936534 PMC3732235

[B24] Demers M. M. McNevin N. Azar N. R. (2013). ADHD and motor control: a review of the motor control deficiencies associated with attention deficit/hyperactivity disorder and current treatment options. Crit. Rev. Phys. Rehabil. Med. 25, 3–4. 10.1615/CritRevPhysRehabilMed.2013009763

[B25] Desain P. Honing H. (1999). Computational models of beat induction: the rule-based approach. J. New Music Res. 28, 29–42. 10.1076/jnmr.28.1.29.3123

[B26] DiMaio S. Grizenko N. Joober R. (2003). Dopamine genes and attention-deficit hyperactivity disorder: a review. J. Psychiatry Neurosci. 28, 27–38. 12587848 PMC161723

[B27] Drew M. R. Simpson E. H. Kellendonk C. Herzberg W. G. Lipatova O. Fairhurst S.. (2007). Transient overexpression of striatal D2 receptors impairs operant motivation and interval timing. J. Neurosci. 27, 7731–7739. 10.1523/JNEUROSCI.1736-07.2007 17634367 PMC6672869

[B28] Durston S. van Belle J. de Zeeuw P. (2011). Differentiating frontostriatal and fronto-cerebellar circuits in attention-deficit/hyperactivity disorder. Biol. Psychiatry 69, 1178–1184. 10.1016/j.biopsych.2010.07.037 20965496

[B29] Emanuele E. Boso M. Cassola F. Broglia D. Bonoldi I. Mancini L.. (2010). Increased dopamine DRD4 receptor mRNA expression in lymphocytes of musicians and autistic individuals: bridging the music-autism connection. Neuro Endocrinol. Lett. 31, 122–125. 20150884

[B30] Engel A. K. Fries P. Singer W. (2001). Dynamic predictions: oscillations and synchrony in top-down processing. Nat. Rev. Neurosci. 2, 704–716. 10.1038/35094565 11584308

[B31] Fair D. A. Bathula D. Nikolas M. A. Nigg J. T. (2012). Distinct neuropsychological subgroups in typically developing youth inform heterogeneity in children with ADHD. Proc. Natl. Acad. Sci. U.S.A. 109, 6769–6774. 10.1073/pnas.1115365109 22474392 PMC3340031

[B32] Fujioka T. Trainor L. J. Large E. W. Ross B. (2009). Beta and gamma rhythms in human auditory cortex during musical beat processing. Ann. N. Y. Acad. Sci. 1169, 89–92. 10.1111/j.1749-6632.2009.04779.x 19673759

[B33] Giedd J. N. Lenroot R. K. Shaw P. Lalonde F. Celano M. White S.. (2008). Trajectories of anatomic brain development as a phenotype. Novartis Found. Symp. 289, 101–112; discussion: 112–108; 193–105. 18497098 10.1002/9780470751251.ch9 PMC3024856

[B34] Grahn J. A. (2012). Neural mechanisms of rhythm perception: current findings and future perspectives. Top. Cogn. Sci. 4, 585–606. 10.1111/j.1756-8765.2012.01213.x 22811317

[B35] Grahn J. A. Brett M. (2007). Rhythm and beat perception in motor areas of the brain. J. Cogn. Neurosci. 19, 893–906. 10.1162/jocn.2007.19.5.893 17488212

[B36] Grahn J. A. Brett M. (2009). Impairment of beat-based rhythm discrimination in Parkinson's disease. Cortex 45, 54–61. 10.1016/j.cortex.2008.01.005 19027895

[B37] Grahn J. A. Rowe J. B. (2009). Feeling the beat: premotor and striatal interactions in musicians and nonmusicians during beat perception. J. Neurosci. 29, 7540–7548. 10.1523/JNEUROSCI.2018-08.2009 19515922 PMC2702750

[B38] Graybiel A. M. (1997). The basal ganglia and cognitive pattern generators. Schizophr. Bull. 23, 459–469. 10.1093/schbul/23.3.459 9327509

[B39] Grube M. Lee K.-H. Griffiths T. D. Barker A. T. Woodruff P. W. (2010). Frontiers: transcranial magnetic theta-burst stimulation of the human cerebellum distinguishes absolute, duration-based from relative, beat-based perception of subsecond time intervals. Front. Auditory Cogn. Neurosci. 1:171 10.3389/fpsyg.2010.00171 PMC3153783 21833234

[B40] Hove M. J. Gravel N. Spencer R. M. C. Valera E. M. (2017). Finger tapping and pre-attentive sensorimotor timing in adults with ADHD. Exp. Brain Res. 235, 3663–3672. 10.1007/s00221-017-5089-y 28913612 PMC5671889

[B41] Huron D. B. (2006). Sweet Anticipation: Music and the Psychology of Expectation. Cambridge, MA: MIT press.

[B42] Iversen J. R. Repp B. H. Patel A. D. (2009). Top-down control of rhythm perception modulates early auditory responses. Ann. N. Y. Acad. Sci. 1169, 58–73. 10.1111/j.1749-6632.2009.04579.x 19673755

[B43] Kaiser M.-L. Schoemaker M. Albaret J.-M. Geuze R. (2015). What is the evidence of impaired motor skills and motor control among children with attention deficit hyperactivity disorder (ADHD)? Systematic review of the literature. Res. Dev. Disabil. 36, 338–357. 10.1016/j.ridd.2014.09.023 25462494

[B44] Kebir O. Joober R. (2011). Neuropsychological endophenotypes in attention-deficit/hyperactivity disorder: a review of genetic association studies. Eur. Arch. Psychiatry Clin. Neurosci. 261, 583–594. 10.1007/s00406-011-0207-5 21409419

[B45] Keller P. E. Rieger M. (2009). Special issue—Musical movement and synchronization. Music Percept. 26, 397–400. 10.1525/mp.2009.26.5.397

[B46] Krause V. Schnitzler A. Pollok B. (2010). Functional network interactions during sensorimotor synchronization in musicians and non-musicians. Neuroimage 52, 245–251. 10.1016/j.neuroimage.2010.03.081 20363337

[B47] Large E. W. Herrera J. A. Velasco M. J. (2015). Neural networks for beat perception in musical rhythm. Front. Syst. Neurosci. 9:159. 10.3389/fnsys.2015.00159 26635549 PMC4658578

[B48] Large E. W. Kolen J. F. (1994). Resonance and the perception of musical meter. Conn. Sci. 6, 177–208. 10.1080/09540099408915723

[B49] Large E. W. Palmer C. (2002). Perceiving temporal regularity in music. Cogn. Sci. 26, 1–37. 10.1207/s15516709cog2601_1

[B50] Large E. W. Snyder J. S. (2009). Pulse and meter as neural resonance. Ann. N. Y. Acad. Sci. 1169, 46–57. 10.1111/j.1749-6632.2009.04550.x 19673754

[B51] Leisman G. Melillo R. (2010). Effects of motor sequence training on attentional performance in ADHD children. Int. J. Disabil. Hum. Dev. 9, 275–282. 10.1515/IJDHD.2010.043

[B52] Levitin D. J. Chordia P. Menon V. (2012). Musical rhythm spectra from Bach to Joplin obey a 1/f power law. Proc. Natl. Acad. Sci. U.S.A. 109, 3716–3720. 10.1073/pnas.1113828109 22355125 PMC3309746

[B53] Linkenkaer-Hansen K. Nikouline V. V. Palva J. M. Ilmoniemi R. J. (2001). Long-range temporal correlations and scaling behavior in human brain oscillations. J. Neurosci. 21, 1370–1377. 10.1523/JNEUROSCI.21-04-01370.2001 11160408 PMC6762238

[B54] Longuet-Higgins H. C. Lee C. S. (1982). The perception of musical rhythms. Perception 11, 115–128. 10.1068/p110115 7155765

[B55] Loo S. K. McGough J. J. McCracken J. T. Smalley S. L. (2017). Parsing heterogeneity in attention-deficit hyperactivity disorder using EEG-based subgroups. J. Child Psychol. Psychiatry 59, 223–231. 10.1111/jcpp.12814 28921526 PMC5812789

[B56] Mazaheri A. Fassbender C. Coffey-Corina S. Hartanto T. A. Schweitzer J. B. Mangun G. R. (2014). Differential oscillatory electroencephalogram between attention-deficit/hyperactivity disorder subtypes and typically developing adolescents. Biol. Psychiatry 76, 422–429. 10.1016/j.biopsych.2013.08.023 24120092 PMC3972379

[B57] McAuley J. D. (2010). Tempo and rhythm, in Music Perception. Springer Handbook of Auditory Research, Vol. 36, eds Jones M. R. Fay R. R. Popper A. N. (New York, NY: Springer Science + Business Media), 165–199.

[B58] Merchant H. Grahn J. A. Trainor L. Rohrmeier M. Fitch W. T. (2015). Finding the beat: a neural perspective across humans and non-human primates. Philos. Trans. R. Soc. B Biol. Sci. 370:20140093. 10.1098/rstb.2014.0093 25646516 PMC4321134

[B59] Moreno S. Bialystok E. Barac R. Schellenberg E. G. Cepeda N. J. Chau T. (2011). Short-term music training enhances verbal intelligence and executive function. Psychol. Sci. 22, 1425–1433. 10.1177/0956797611416999 21969312 PMC3449320

[B60] Mueller A. Hong D. S. Shepard S. Moore T. (2017). Linking ADHD to the neural circuitry of attention. Trends Cogn. Sci. 21, 474–488. 10.1016/j.tics.2017.03.009 28483638 PMC5497785

[B61] Müller V. Sänger J. Lindenberger U. (2013). Intra- and Inter-Brain Synchronization during Musical Improvisation on the Guitar. PLoS ONE 8:e73852. 10.1371/journal.pone.0073852 24040094 PMC3769391

[B62] Nigg J. T. Willcutt E. G. Doyle A. E. Sonuga-Barke E. J. (2005). Causal heterogeneity in attention-deficit/hyperactivity disorder: do we need neuropsychologically impaired subtypes? Biol. Psychiatry 57, 1224–1230. 10.1016/j.biopsych.2004.08.025 15949992

[B63] Nikolas M. A. Nigg J. T. (2015). Moderators of neuropsychological mechanism in attention-deficit hyperactivity disorder. J. Abnorm. Child Psychol. 43, 271–281. 10.1007/s10802-014-9904-7 25037459 PMC4300292

[B64] Noreika V. Falter C. M. Rubia K. (2013). Timing deficits in attention-deficit/hyperactivity disorder (ADHD): evidence from neurocognitive and neuroimaging studies. Neuropsychologia 51, 235–266. 10.1016/j.neuropsychologia.2012.09.036 23022430

[B65] Nozaradan S. (2014). Exploring how musical rhythm entrains brain activity with electroencephalogram frequency-tagging. Philos. Trans. R. Soc. B Biol. Sci. 369:20130393. 10.1098/rstb.2013.0393 25385771 PMC4240960

[B66] Nozaradan S. Peretz I. Mouraux A. (2012). Selective neuronal entrainment to the beat and meter embedded in a musical rhythm. J. Neurosci. 32, 17572–17581. 10.1523/JNEUROSCI.3203-12.2012 23223281 PMC6621650

[B67] Nozaradan S. Schwartze M. Obermeier C. Kotz S. A. (2017). Specific contributions of basal ganglia and cerebellum to the neural tracking of rhythm. Cortex 95, 156–168. 10.1016/j.cortex.2017.08.015 28910668

[B68] Palmer C. (1997). Music performance. Annu. Rev. Psychol. 48, 115–138. 10.1146/annurev.psych.48.1.115 9046557

[B69] Patel A. D. Iversen J. R. (2014). The evolutionary neuroscience of musical beat perception: the Action Simulation for Auditory Prediction (ASAP) hypothesis. Front. Syst. Neurosci. 8:57. 10.3389/fnsys.2014.00057 24860439 PMC4026735

[B70] Patel A. D. Iversen J. R. Bregman M. R. Schulz I. (2009). Studying synchronization to a musical beat in nonhuman animals. Ann. N. Y. Acad. Sci. 1169, 459–469. 10.1111/j.1749-6632.2009.04581.x 19673824

[B71] Phillips-Silver J. Trainor L. J. (2005). Feeling the beat: movement influences infant rhythm perception. Science 308, 1430–1430. 10.1126/science.1110922 15933193

[B72] Phillips-Silver J. Trainor L. J. (2007). Hearing what the body feels: auditory encoding of rhythmic movement. Cognition 105, 533–546. 10.1016/j.cognition.2006.11.006 17196580

[B73] Povel D.-J. Essens P. (1985). Perception of temporal patterns. Music Percept. 2, 411–440. 10.2307/40285311 3991313

[B74] Puyjarinet F. Bégel V. Lopez R. Dellacherie D. Dalla Bella S. (2017). Children and adults with Attention-Deficit/Hyperactivity Disorder cannot move to the beat. Sci. Rep. 7, 11550. 10.1038/s41598-017-11295-w 28912422 PMC5599521

[B75] Quintana J. Fuster J. M. (1999). From perception to action: temporal integrative functions of prefrontal and parietal neurons. Cerebral Cortex 9, 213–221. 10.1093/cercor/9.3.213 10355901

[B76] Raichle M. E. (2010). Two views of brain function. Trends Cogn. Sci. 14, 180–190. 10.1016/j.tics.2010.01.008 20206576

[B77] Rammsayer T. Altenmüller E. (2006). Temporal information processing in musicians and nonmusicians. Music Percept. 24, 37–48. 10.1525/mp.2006.24.1.37

[B78] Rankin S. K. Fink P. W. Large E. W. (2014). Fractal structure enables temporal prediction in music. J. Acoust. Soc. Am. 136, EL256–EL262. 10.1121/1.4890198 25324107

[B79] Rankin S. K. Large E. W. Fink P. W. (2009). Fractal tempo fluctuation and pulse prediction. Music Percept. 26, 401–413. 10.1525/mp.2009.26.5.401 25190901 PMC4151502

[B80] Repp B. H. (1995). Expressive timing in Schumann's “Träumerei:”An analysis of performances by graduate student pianists. J. Acoust. Soc. Am. 98, 2413–2427. 7593927 10.1121/1.413276

[B81] Repp B. H. Su Y. -H. (2013). Sensorimotor synchronization: a review of recent research (2006–2012). Psychon. Bull. Rev. 20, 403–452. 10.3758/s13423-012-0371-2 23397235

[B82] Roden I. Grube D. Bongard S. Kreutz G. (2014). Does music training enhance working memory performance? Findings from a quasi-experimental longitudinal study. Psychol. Music 42, 284–298. 10.1177/0305735612471239

[B83] Rommelse N. N. Altink M. E. Oosterlaan J. Beem L. Buschgens C. J. Buitelaar J.. (2008). Speed, variability, and timing of motor output in ADHD: which measures are useful for endophenotypic research? Behav. Genet. 38, 121–132. 10.1007/s10519-007-9186-8 18071893 PMC2257997

[B84] Rubia K. (2016). Can functional decoding elucidate meta-analytic brain dysfunctions in adult attention-deficit/hyperactivity disorder? Biol. Psychiatry 80, 890–892. 10.1016/j.biopsych.2016.10.003 27839558

[B85] Salimpoor V. N. Zatorre R. J. (2013). Neural interactions that give rise to musical pleasure. Psychol. Aesthet. Creat. Arts 7, 62 10.1037/a0031819

[B86] Schlaug G. (2015). Musicians and music making as a model for the study of brain plasticity, in Music, Neurology, and Neuroscience: Evolution, the Musical Brain, Medical Conditions, and Therapies, Progress in Brain Research, Vol. 217, eds Altenmüller E. Finger S. Boller F. (Amsterdam: Elsevier) 37–55. 10.1016/bs.pbr.2014.11.020 PMC4430083 25725909

[B87] Schroeder C. E. Wilson D. A. Radman T. Scharfman H. Lakatos P. (2010). Dynamics of active sensing and perceptual selection. Curr. Opin. Neurobiol. 20, 172–176. 10.1016/j.conb.2010.02.010 20307966 PMC2963579

[B88] Schubotz R. I. (2007). Prediction of external events with our motor system: towards a new framework. Trends Cogn. Sci. 11, 211–218. 10.1016/j.tics.2007.02.006 17383218

[B89] Schultz W. (1998). Predictive reward signal of dopamine neurons. J. Neurophysiol. 80, 1–27. 10.1152/jn.1998.80.1.1 9658025

[B90] Schultz W. (2007). Multiple Dopamine Functions at Different Time Courses. Annu. Rev. Neurosci. 30, 259–288. 10.1146/annurev.neuro.28.061604.135722 17600522

[B91] Schwartze M. Kotz S. A. (2013). A dual-pathway neural architecture for specific temporal prediction. Neurosci. Biobehav. Rev. 37, 2587–2596. 10.1016/j.neubiorev.2013.08.005 23994272

[B92] Shaffer R. J. Jacokes L. E. Cassily J. F. Greenspan S. I. Tuchman R. F. Stemmer P. J. (2001). Effect of Interactive Metronome® training on children with ADHD. Am. J. Occupat. Ther. 55, 155–162. 10.5014/ajot.55.2.155 11761130

[B93] Shaw P. Gornick M. Lerch J. Addington A. Seal J. Greenstein D.. (2007). Polymorphisms of the dopamine D4 receptor, clinical outcome, and cortical structure in attention-deficit/hyperactivity disorder. Arch. Gen. Psychiatry 64, 921–931. 10.1001/archpsyc.64.8.921 17679637

[B94] Silberstein R. B. Pipingas A. Farrow M. Levy F. Stough C. K. Camfield D. A. (2016). Brain functional connectivity abnormalities in attention-deficit hyperactivity disorder. Brain Behav. 6:e00583. 10.1002/brb3.583 28032006 PMC5167009

[B95] Silk T. J. Vance A. Rinehart N. Bradshaw J. L. Cunnington R. (2009). White-matter abnormalities in attention deficit hyperactivity disorder: a diffusion tensor imaging study. Hum. Brain Mapp. 30, 2757–2765. 10.1002/hbm.20703 19107752 PMC6870653

[B96] Slater J. Ashley R. Tierney A. Kraus N. (2018). Got rhythm? Better inhibitory control is linked with more consistent drumming and enhanced neural tracking of the musical beat in adult percussionists and nonpercussionists. J. Cogn. Neurosci. 30, 14–24. 10.1162/jocn_a_01189 28949825

[B97] Slater J. Azem A. Nicol T. Swedenborg B. Kraus N. (2017). Variations on the theme of musical expertise: cognitive and sensory processing in percussionists, vocalists and non-musicians. Eur. J. Neurosci. 45, 952–963. 10.1111/ejn.13535 28177157 PMC5378620

[B98] Smit D. J. Linkenkaer-Hansen K. de Geus E. J. (2013). Long-range temporal correlations in resting-state alpha oscillations predict human timing-error dynamics. J. Neurosci. 33, 11212–11220. 10.1523/jneurosci.2816-12.2013 23825424 PMC6618606

[B99] Smit D. J. A. Anokhin A. P. (2017). Development and genetics of brain temporal stability related to attention problems in adolescent twins. Int. J. Psychophysiol. 115, 86–97. 10.1016/j.ijpsycho.2016.07.498 27418541

[B100] Smith A. Taylor E. Rogers J. W. Newman S. Rubia K. (2002). Evidence for a pure time perception deficit in children with ADHD. J. Child Psychol. Psychiatry 43, 529–542. 10.1111/1469-7610.00043 12030598

[B101] Snyder J. S. Large E. W. (2005). Gamma-band activity reflects the metric structure of rhythmic tone sequences. Cogn. Brain Res. 24, 117–126. 10.1016/j.cogbrainres.2004.12.014 15922164

[B102] Sumbre G. Muto A. Baier H. Poo M. M. (2008). Entrained rhythmic activities of neuronal ensembles as perceptual memory of time interval. Nature 456, 102. 10.1038/nature07351 18923391 PMC2896960

[B103] Swanson J. Flodman P. Kennedy J. Spence M. A. Moyzis R. Schuck S.. (2000). Dopamine genes and ADHD. Neurosci. Biobehav. Rev. 24, 21–25. 10.1016/S0149-7634(99)00062-7 10654656

[B104] Tarr B. Launay J. Dunbar R. I. (2014). Music and social bonding:“self-other” merging and neurohormonal mechanisms. Front. Psychol. 5:1096. 10.3389/fpsyg.2014.01096 25324805 PMC4179700

[B105] Tierney A. Kraus N. (2013). Neural responses to sounds presented on and off the beat of ecologically valid music. Front. Syst. Neurosci. 7:14. 10.3389/fnsys.2013.00014 23717268 PMC3650712

[B106] Tierney A. Kraus N. (2015). Neural entrainment to the rhythmic structure of music. J. Cogn. Neurosci. 27, 400–408. 10.1162/jocn_a_00704 25170794

[B107] Toplak M. E. Dockstader C. Tannock R. (2006). Temporal information processing in ADHD: findings to date and new methods. J. Neurosci. Methods 151, 15–29. 10.1016/j.jneumeth.2005.09.018 16378641

[B108] Trainor L. J. Shahin A. J. Roberts L. E. (2009). Understanding the benefits of musical training: effects on oscillatory brain activity. Ann. N. Y. Acad. Sci. 1169, 133–142. 10.1111/j.1749-6632.2009.04589.x 19673769

[B109] Tye C. McLoughlin G. Kuntsi J. Asherson P. (2011). Electrophysiological markers of genetic risk for attention deficit hyperactivity disorder. Expert Rev. Mol. Med. 13, e9–e9. 10.1017/S1462399411001797 21426626 PMC4042910

[B110] Uhlhaas P. J. Roux F. Rodriguez E. Rotarska-Jagiela A. Singer W. (2010). Neural synchrony and the development of cortical networks. Trends Cogn. Sci. 14, 72–80. 10.1016/j.tics.2009.12.002 20080054

[B111] USDHHS (2013). US Department of Health and Human Services. FDA approval letter for NEBA System. Available online at: http://www.accessdata.fda.gov/cdrh_docs/pdf11/K112711.pdf

[B112] Valera E. M. Spencer R. M. Zeffiro T. A. Makris N. Spencer T. J. Faraone S. V.. (2010). Neural substrates of impaired sensorimotor timing in adult attention-deficit/hyperactivity disorder. Biol. Psychiatry 68, 359–367. 10.1016/j.biopsych.2010.05.012 20619827 PMC2917236

[B113] Wallentin M. Nielsen A. H. Friis-Olivarius M. Vuust C. Vuust P. (2010). The musical ear test, a new reliable test for measuring musical competence. Learn. Individ. Differ. 20, 188–196. 10.1016/j.lindif.2010.02.004

[B114] Wiener M. Lohoff F. W. Coslett H. B. (2011). Double dissociation of dopamine genes and timing in humans. J. Cogn. Neurosci. 23, 2811–2821. 10.1162/jocn.2011.21626 21261454

[B115] Zatorre R. J. Chen J. L. Penhune V. B. (2007). When the brain plays music: auditory–motor interactions in music perception and production. Nat. Rev. Neurosci. 8, 547–558. 10.1038/nrn2152 17585307

[B116] Zelaznik H. N. Vaughn A. J. Green J. T. Smith A. L. Hoza B. Linnea K. (2012). Motor timing deficits in children with attention-deficit/hyperactivity disorder. Hum. Mov. Sci. 31, 255–265. 10.1016/j.humov.2011.05.003 21852012 PMC3223335

